# Genetic diversity of Morato's Digger Toad, *Proceratophrys moratoi*: spatial structure, gene flow, effective size and the need for differential management strategies of populations

**DOI:** 10.1590/1678-4685-gmb-2016-0025

**Published:** 2017-06-05

**Authors:** Mauricio P. Arruda, William P. Costa, Shirlei M. Recco-Pimentel

**Affiliations:** 1 Departamento de Biologia Estrutural e Funcional, Instituto de Biologia, Universidade Estadual de Campinas (UNICAMP), Campinas, SP, Brazil; 2 Laboratório de Biologia, Instituto Federal de Educação, Ciência e Tecnologia do Amazonas (IFAM), Tabatinga, AM, Brazil

**Keywords:** Proceratophrys moratoi, microsatellite, population structure, management unit, conservation

## Abstract

The Morato's Digger Toad, *Proceratophrys moratoi*, is a critically endangered toad species with a marked population decline in southern Brazilian Cerrado. Despite this, new populations are being discovered, primarily in the northern part of the distribution range, which raises a number of questions with regard to the conservation status of the species. The present study analyzed the genetic diversity of the species based on microsatellite markers. Our findings permitted the identification of two distinct management units. We found profound genetic structuring between the southern populations, on the left margin of the Tietê River, and all other populations. A marked reduction was observed in the contemporary gene flow among the central populations that are most affected by anthropogenic impacts, such as extensive sugar cane plantations, which presumably decreases habitat connectivity. The results indicated reduced diversity in the southern populations which, combined with a smaller effective population size, may make these populations more susceptible to extinction. We recommend the reclassification of *P. moratoi* as vulnerable and the establishment of a special protection program for the southern populations. Our results provide important insights about the local extinction of southern populations of this toad.

## Introduction

The understanding of genetic diversity, population structure, gene flow, and the dispersal capacity of endangered species are fundamental to conservation genetics (Beebee, 2005). Studies of this kind are especially important for elucidating population decline, the definition of conservation status, and the development of management strategies (Beebee, 2005; Funk *et al.*, 2012).

The Morato's Digger Toad, *Proceratophrys moratoi* (Jim and Caramaschi, 1980), is a small digging toad which is found generally in “campo sujo” habitats (savanna with some shrubs) of the Cerrado biome, often in the proximity of streams (Jim and Caramaschi, 1980). The Cerrado is a biodiversity “hotspot” which has suffered high rates of habitat loss, with only 21% of the original vegetation remaining intact (Conservation International, 2011) most of which now are small, isolated fragments (Mittermeier *et al.*, 2005).

Due to its restricted geographic distribution and the evidence of decline and extinction in the southern populations, the species is listed by the IUCN as critically endangered (Cruz and Caramaschi, 2004). However, recent studies have recorded a number of new occurrence localities, especially in the northern part of its original distribution, which have led to considerable debate on its conservation status (*e.g.* Brasileiro *et al.*, 2008; Carvalho Jr *et al.*, 2010; Rolim *et al.*, 2010; Martins and Giaretta, 2012).

The susceptibility of *P. moratoi* to anthropogenic impacts is uncertain. The population of Botucatu, the type locality of the species, has been considered to be extinct since 1998, since when no more individuals have been found, despite numerous field studies at this locality and surrounding areas during the rainy season (Rolim, 2009). One possible cause of this disappearance is habitat destruction related to farming and urban development, with remaining populations being restricted to protected areas (Brasileiro *et al.*, 2008), such as Brotas, which is located on the right margin of the Tietê river. However, one of the known populations, São Carlos, is located in an area of intense agricultural activity, indicating that the species may be able to tolerate a certain level of human impact or even colonize modified habitats (Carvalho Jr *et al.*, 2010). In fact, little is known about the natural history of *P. moratoi* or its long-term capacity for survival in degraded habitats.

Based on the analysis of morphological, cytogenetic, and morphometric characteristics, Costa (2012) detected reduced levels of inter-population divergence, although it was possible to identify patterns of variation in coloration and some morphometric traits, such as body size, which permitted the differentiation of populations. However, Forti *et al.* (2016) observed low variation in the traits of calls and a high level of haplotype sharing among populations (in the 16S mitochondrial gene).

To complement these findings, we analyzed the genetic characteristics of *P. moratoi* populations, using microsatellite markers, in order to i) estimate the genetic diversity, inbreeding levels, and effective size of populations; ii) characterize the spatial genetic structure and gene flow between populations; and iii) integrate the results into management recommendations for the conservation of this species.

## Materials and Methods

### Study area and ethics statement

**Figure 1:**
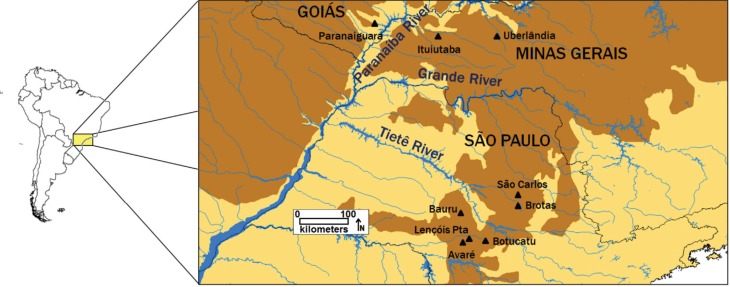
Localities analyzed. Localities sampled during the present study, located within the Cerrado biome (brown shading) in relation to the major local rivers, the Paranaíba, Grande, and Tietê. Shown are the original limits of the Cerrado biome, that is, prior to recent anthropogenic impacts.

All the areas in which *P. moratoi* populations were recorded prior to 2013 were surveyed (Figure 1). Fieldwork was conducted from October to March (rainy season) in the years between 2010 and 2013. Samples were collected in the field using two methods to capture toads: acoustic and visual survey at breeding sites, as proposed by Scott Jr and Woodward (1994), and pitfall traps with drift fence (Cechin and Martins, 2000).

The sampling was non-destructive with no impact on endangered populations of *P. moratoi*. Saliva samples were collected from all individuals used in the study. During the course of the study, samples of salivary mucous were obtained from between one and 41 adult individuals from each site (Table 1). The salivary mucous samples were collected using the protocol described by Pidancier *et al.* (2003), with modification, where following capture, each animal's oral cavity was opened gently by depressing the lower part of the mandible with a thumb. A commercially-available sterile cotton swab was then introduced into the oral cavity and wiped gently around the internal surface of the cavity using circular movements for approximately 2 min, until the saliva sample was obtained. The swab was then stored in a micro-tube containing 1 mL of lysis buffer (10 mM tris-HCl pH 8.0, 1 mM EDTA pH 8.0, 100 mM NaCl, 2% SDS) and kept on ice until storage in a freezer at −20 °C. After saliva collection, all captured individuals were released back into the wild without being marked in any way.

**Table 1:** Basic characteristics of the *P. moratoi* study populations, grouped by geographic region (see Figure 1) and levels of intrapopulation genetic diversity.

Region	Population-state	Latitude	Longitude	*n* ^1^	P	N_a_	N_e_	AR	H_o_	H_e_	pHWE	*F*_*IS*_
North	1-Paranaiguara-GO^2^	-18.76°	-50.61°	3	59%	2.47	2.27	1.48	0.45	0.48	0.994	0.080
	2-Ituiutaba-MG	-19.01°	-49.45°	4	88%	2.35	1.85	1.45	0.35	0.45	0.378	0.242
	3-Uberlândia-MG	-19.00°	-48.32°	12	82%	3.65	2.36	1.39	0.33	0.39	0.449	0.148
Central	4-São Carlos-SP	-22.02°	-47.94°	40	88%	4.24	2.47	1.42	0.40	0.42	0.142	0.043
	5-Brotas-SP	-22.21°	-47.91°	41	88%	4.53	2.65	1.41	0.37	0.41	0.007	0.102
South	6-Bauru-SP	-22.35°	-49.02°	24	65%	3.41	2.21	1.38	0.38	0.38	0.481	-0.008
	7-Avaré-SP	-22.89°	-48.95°	3	53%	1.76	1.50	1.28	0.26	0.28	0.602	0.100
	8-Lençóis Paulista-SP	-22.82°	-48.88°	1	24%	1.24	1.24	1.23	0.24	0.24	-	-
	9-Botucatu-SP	-22.89°	-48.51°	13	70%	2.10	1.81	-	0.19	0.39	0.028	0.573
	MD^3^			141^4^	80%	3.77	2.36	1.40	0.36	0.40	0.211	0.117

1 *n* = sample size (individuals); P = percentage of polymorphic loci; N_a_ = mean number of alleles per locus; N_e_ = number of effective alleles; AR = allelic richness; H_o_ = observed heterozygosity; H_e_ = expected heterozygosity; pHWE = probability of deviations from Hardy-Weinberg Equilibrium (p values lower than 0.003, with the Bonferroni correction, are considered to be significant); *F*_*IS*_ = coefficient of endogamy;

2 GO = Goiás; MG = Minas Gerais; SP = São Paulo;

3 MD = mean value considering the populations separately according to their respective sample sizes (*n*);

4 Total.

Amphibians were captured within a maximum radius of 500 meters from the coordinates of each site (see Table 1). The reduced sample size for some populations was due to the scarcity of calling adults at local sites, despite repeated visits by collectors during the rainy season. Given this, and considering the unique situation of the species, with numerous declining and extinct populations, it was deemed necessary to conduct the analysis, in spite of the small size of some samples. To avoid a possible recapture bias in the population analyses, whenever identical genotypes were identified in the combined analysis of all loci in CERVUS 3.0.3 (Marshall *et al.*, 1998), only one of the individuals was included in the population analyses.

### Extraction of genomic DNA

A modified protocol based on Pidancier *et al.* (2003) was used to obtain samples from salivary mucous. This is a non-destructive method for the extraction of genomic DNA. The extracted DNA was diluted in a solution of Tris-EDTA (10 mM Tris-HCl pH 8.0, 1 mM EDTA pH 8.0). Concentration and purity of the DNA were estimated using a NanoDrop™ ND-1000 spectrophotometer.

The first population of the species was identified in the district of Rubião Júnior (municipality of Botucatu, SP) and according to Rolim (2009), this population is now extinct. Individuals had been collected from this population in 1976, fixed in formalin, and deposited in the amphibian collection at UNESP in Botucatu. For the analysis of this population, samples of muscle tissue were extracted from 13 specimens, and the DNA was extracted using the modification procedure described by Boom *et al.* (1990), with modifications. Initially, the tissue was diluted in a pH 8.0 buffer (Tris-HCl 10 mM) for 20 min, and after maceration, it was transferred to 500 μL of pH 6.4 lysis buffer (guanidine thiocyanate 4M, Tris-HCl 10 mM, EDTA 20 mM and Triton X-100 0.65%). The solution was then incubated overnight at 60 °C, followed by the addition of 500 μL of chloroform. After inversion, the suspension was centrifuged for 3 min at 3,824 rcf. Approximately 450 μL of the supernatant was transferred to a new microtube, to which 1 mL of lysis buffer and 40 μL of silica particles were added for 10 min, and the suspension was then centrifuged for 3 min at 3,824 rcf. The supernatant (1 mL) was discarded, 1 mL of washing buffer (guanidine thiocyanate 4 M, Tris-HCl 10 mM pH 6.4) was added and centrifuged for 3 min at 3,824 rcf. The supernatant was again discarded (1 mL) and the solution was topped off with freezing absolute ethanol. The micro-tube was inverted repeatedly, centrifuged at 17,949 rcf for 15 min, and after the ethanol was discarded, the pellet was dried and the DNA was diluted in 110 μL of TE buffer (10 mM Tris-HCl pH 8.0; 1 mM EDTA) and stored at −20 °C.

### Genotyping of the populations and evaluation of the microsatellite markers

The 22 microsatellite loci developed by Arruda *et al.* (2012) were used to genotype the *P. moratoi* populations. PCR assays prepared and amplifications were done following the recommendations of these authors. Successfully amplified PCR products were analyzed in a Dual Dedicated Height vertical electrophoresis sequencing system (CBS Scientific) using a denaturing 6% polyacrylamide gel and stained with silver nitrate (Creste *et al.*, 2001). Allele size was estimated in GELANALYZER 2010A (Lazar and Lazar, 2010).

Only the markers that presented a clear profile (without unspecific or stutter bands) and had been amplified in at least one individual from each population (except for Botucatu, given the loss of genotypes due to the degradation of the samples by the formalin – see Results) were used for the population-level analyses presented here. The markers were evaluated in MICRO-CHECKER 2.2.3 (van Oosterhout *et al.*, 2004) for the identification of genotyping errors and evidence of null alleles, using the Brookfield (1996) method with 1,000 randomizations and Bonferroni correction (Rice, 1989). Given the assumptions of this method, this analysis included only the populations with samples of at least 20 individuals. The potential effect of null alleles on genetic differentiation was estimated in FreeNA (Chapuis and Estoup, 2007) with 100 replicates using the excluding null alleles “ENA” method to adjust the genotypes. Potential genotype linkage disequilibrium was evaluated in GENEPOP 4.1.0 (Rousset, 2008), with 10,000 dememorization steps and 100 batches of 5,000 interactions per batches.

### Population analysis

Intra-population variability was quantified according to the observed (H_o_) and expected heterozygosity (H_e_), the percentage of polymorphic loci (P), number of alleles (N_a_), and the effective number of alleles (N_e_) (Hartl and Clark, 1989), all of which were estimated in POPGENE 1.32 (Yeh *et al.*, 1997). The intra-population inbreeding coefficient (*Fis*) was calculated in FSTAT 2.9.3.2 (Goudet, 1997), as were allelic richness (AR) (except for the Botucatu population, for which not all the loci were amplified), corrected by the minimum sample size of one individual, and the divergent values of allelic richness among groups of samples were examined using permutation test with 15,000 permutations. The populations were also examined for possible deviations from Hardy-Weinberg equilibrium using GENEPOP with 10,000 dememorization steps and 100 batches of 5,000 interactions per batches with Bonferroni correction.

Inferences on population structure may be affected by deviations from neutrality, such as outlier markers that are suffering selection pressure, resulting in certain biases in the analyses. The LOSITAN (Antao *et al.*, 2008) application was used to identify such loci, with runs of 200,000 simulations using an initial run to identify a set of neutral markers, based on two mutation models, the stepwise mutation model and the infinite alleles model. Neutral markers, *i.e.*, non-outliers, were included in all the subsequent analyses.

Levels of differentiation in the population were evaluated in ARLEQUIN 3.5.1.2 (Excoffier and Schneider, 2006), based on the *F* statistic, with the *F*_*ST*_ and *R*_*ST*_ indices. The latter was adjusted to the stepwise mutation model of evolution for microsatellite markers (Slatkin, 1995), using 10,000 permutations. The permutation test, run in SPAGeDI 1.4c (Hardy and Vekemans, 2002), was used to verify the contribution of gradual mutations to the genetic structuring. The number of private alleles was calculated in CONVERT 1.3 (Glaubitz, 2004). The number of genetically distinct samples clusters (*k*) was determined by a Bayesian approach using STRUCTURE 2.3.4 (Pritchard *et al.*, 2000), with five runs conducted with 1,000,000 replicates and a burn-in of 200,000, using the admixture model and the frequency of correlated alleles, without prior population information, with between one and 10 clusters (*k*) and the most probable value being selected by the Evanno method in STRUCTURE HARVESTER 0.6.93 (Earl and von Holdt, 2012). The STRUCTURE software also permitted to calculate the proportional contribution of each individual to the clusters (Q – membership coefficient).

Spatial discontinuities in gene flow were evaluated by Bayesian clustering with a Hidden Gaussian Random Field (HGRF), run in TESS 2.3.1 (Chen *et al.*, 2007), using the mixed conditional auto-regressive model (CAR). Ten independent runs were conducted for each *K*_*max*_ cluster (between two and 10 clusters) inferred from the analysis, with 200,000 sweeps, burn-in of 40,000, and the spatial interaction parameter (ψ) fixed at 0.6, as recommended by the authors. To identify the number of sample clusters present in the data, we plotted the deviance information criterion (DIC) produced for the comparison with *K*_*max*_ and selected the value of *K*_*max*_ when the DIC stabilized (Durand *et al.*, 2009) or when the Q matrix of posterior probabilities stabilized (no additional clusters apparent). The CLUMMP 1.12b program (Jakobsson and Rosenberg, 2007) was used to correct possible discrepancies between runs, by analyzing the Q values of the replicates produced by the STRUCTURE and TESS programs. Isolation by distance was used to identify barriers to dispersal of landscape by examining the relationship between the genetic differentiation (*R*_*ST*_ index) and geographic (Euclidean) distance among the sample points, with 10,000 randomizations run in IBD 1.52 (Bohonak, 2002). The protocol of Funk *et al.* (2012) was used to delineate possible management units.

Estimates of contemporary gene flow were obtained in BAYESASS+ 3.0.3 (Wilson and Rannala, 2003). Five independent runs with random seed values were conducted with 10,000,000 iterations and a burn-in of 1,000,000. These demographic analyses based on Bayesian inference tend to fall short when sample size is small. In this case, the analyses were conducted carefully by closely observing the stable probability values defined during the course of the analyses by two different methods (see below). Delta values for the migration rates (*m*_c_), allele frequencies (A), and endogamy (*f*) were adjusted (*m*_c_ = 0.15; A = 0.70; *f* = 0.70) to ensure that around 20–60% of the total changes would be accepted, as recommended by Wilson and Rannala (2003). Convergence was also measured by examining the log-probability in the TRACER 1.5 program (Rambaut and Drummond, 2007) to ensure the proper mixing of parameters. In this program, the formation of a plateau with regular oscillations (*i.e.* no persistent hills and valleys) indicates good mixing and effective sampling from the posterior distribution.

Mean long-term rates of migration (M) were estimated in MIGRATE-n 3.6 (Beerli and Felsenstein, 2001), which was approximately 4N_e_ in the past (Beerli, 2004). This rate was estimated by M = *m*_*h*_ / μ, where *m*_*h*_ is the migration rate and μ the mutation rate. This program was also used to estimate effective population size (Θ = 4N_e_μ). A mutation rate of 1.27 x 10^-3^ per generation was considered for the calculation of *m*_*h*_ and N_e_, based on the estimates of Bulut *et al.* (2009) for microsatellites in amphibians. The estimates were based on the Brownian motion mutation model using a constant mutation rate and Bayesian inference with five independent replicates, 3,000 recorded steps, 20 sampling increments, and a burn-in of 400 per chain, as proposed by the Slice distribution, and the prior distribution of Θ (0 100 10) and M (0 1000 100). Contemporary migration (*m*_*c*_) was compared with historical levels (*m*_*h*_) using the Mantel test run in Ztmantel (Bonnet and Van de Peer, 2002), running 10,000 permutations for the evaluation of all the routes analyzed together, and the routes for the northern, central, and southern portions of the geographic range of the species analyzed separately. The tendency for migration over time was evaluated by *m*_*c*_ - *m*_*h*_, where values close to zero indicate equivalent levels of historical and contemporary migration.

The BOTTLENECK 1.2.0.02 program (Cornuet and Luikart, 1996) was used to identify evidence of recent bottlenecks. Initially, a one-tailed signed rank Wilcoxon test was used to verify an excess of heterozygosity (He > Heq) in comparison with that expected in a mutation-drift equilibrium, which is sensitive enough to detect bottlenecks occurring at approximately 2–4 N_e_ generations (Piry *et al.*, 1999), using the mutational model most appropriate for the data, and the two-phase model (TPM) complemented with 70–90% of the stepwise mutation model (SMM). This range encompasses the most probable interval for amphibian microsatellites (Schlötterer, 2000), and variance was set at 12, as recommended by Piry *et al.* (1999). A second test based on the assumption that populations which have passed through bottlenecks are characterized by a reduced proportion of rare alleles (< 10%) due to the loss of these less common alleles, resulting in a “shifted mode distribution”. This test is sensitive enough to detect bottlenecks occurring during the preceding 12 generations (Cornuet and Luikart, 1996), which would coincide with the period of most intense anthropogenic impacts. To guarantee the highest possible statistical power, the tests were conducted only on populations represented by samples of more than 10 individuals, with 10,000 replications.

## Results

### Analysis of the molecular markers

The combined analysis in CERVUS identified identical genotypes (*i.e.* recapture) in only two populations. The highest recapture rate was recorded in Bauru, with three individuals being recaptured (of the total of 27), with 24 samples being used for the analyses. In contrast, only a single pair of identical genotypes was detected in the São Carlos population, with only 40 of the 41 samples being analyzed.

A total of 22 polymorphic loci were evaluated, although one locus, *Pmoratoi*μ5 could not be amplified from the samples obtained from the southern populations, two loci (*Pmoratoi*μ21 and *Pmoratoi*μ24) presented an excess of unspecific bands, nd a further two (*Pmoratoi*μ23 and *Pmoratoi*μ25) had stutter bands, which hampered the interpretation of the genotypes in some populations. In order to avoid biases in the estimates of genetic diversity and population structure, these loci were excluded from the population analyses.

The 17 remaining loci were also amplified in the samples from the Botucatu population. When analyzed in MICRO-CHECKER, no evidence of large allele dropout was found in any of the loci, whereas null alleles were identified in two loci in the Brotas population (*Pmoratoi*μ27 / *r* = 0.078; *Pmoratoi*μ29 / *r* = 0.095) and one in the population from São Carlos (*Pmoratoi*μ29 / *r* = 0.179). Evidence of stuttering was found in one locus from Bauru (*Pmoratoi*μ13 / *r* = 0.103). Overall, significant evidence of genotyping errors was found in only four (7.84%) of the 51 tests (3 populations x 17 loci). The global *F*_*ST*_ values were calculated in the FreeNA application, with the unadjusted value (0.196) being highly similar to the adjusted value (0.194). This led us to maintain these loci in the population analyses, given that small differences support the assumption that the estimates of genetic diversity and population differentiation would not be affected significantly by this degree of genotyping error. No significant linkage disequilibrium was detected between the pairs of loci, based on the analysis in GENEPOP (p > 0.0005, after Bonferroni correction). Given these findings, all 17 independent loci were included in the population analyses.

Using a modified version of the extraction method described by Boom *et al.* (1990), it was possible to recover 15% of the genotypes from the Botucatu samples, that is, it was possible to amplify 34 genotypes from the 221 available (17 loci tested in 13 individuals). At least one individual was amplified in 10 of the loci, and seven of the 13 genotypes were recovered for the loci *Pmoratoi*μ16 and *Pmoratoi*μ29. In contrast, for *Pmoratoi*μ06, *Pmoratoi*μ07, *Pmoratoi*μ08, *Pmoratoi*μ12, *Pmoratoi*μ15, and *Pmoratoi*μ16, all the genotypes were empty. Nevertheless, the population was included in the subsequent analyses.

### Within-population genetic diversity

Estimates of genetic diversity within populations of *P. moratoi* obtained from the analysis of 17 independent loci are presented in Table 1. None of the populations presented a significant deviation from Hardy-Weinberg equilibrium, with the Bonferroni correction.

In the parameters least sensitive to sample size (*i.e.*, AR and H_e_), the highest levels of genetic diversity were recorded for the northern (Paranaiguara, Ituiutaba and Uberlândia) and central (São Carlos e Brotas) populations. By contrast, the southern populations (Bauru, Avaré, Lençóis Paulista and Botucatu) presented the lowest levels of genetic diversity. Based on this finding, allelic richness was compared between the two main groups: group 1 = northern + central populations, and group 2 = southern populations (except Botucatu). This was done in FSTAT using the randomization tests with 1,500 permutation at significance level α = 0.05. Significantly higher genetic diversity was identified in group 1 (p = 0.018 / G1 > G2). While it was not possible to amplify the genotypes in the vast majority of the Botucatu samples, the few recovered genotypes permitted the detection of high levels of endogamy in this extinct population (*F*_*IS*_ = 0.573; see Table 1).

### Analysis of inter-population diversity

The LOSITAN analysis based on the infinite alleles model identified balancing selection in locus *Pmoratoi*μ15 (p = 0.002). Based on the stepwise mutation model, LOSITAN identified the marker *Pmoratoi*μ6 (p = 0.978) as the most likely to have been affected by positive, *i.e.*, directional selection. The permutation test run in SPAGeDI detected a significant contribution (μ > m, p = 0.000) of the stepwise mutations to population structuring, indicating that the *R*_*ST*_ provided the best fit for the data. Based on this result, the most adequate LOSITAN output was that which determined that the locus *Pmoratoi*μ6 is possibly under selection, and for this reason it was excluded from all subsequent analyses.

In the pairwise comparisons using the *R*_*ST*_ index of population differentiation (Table 2) at significance level α = 0.05, the most significant level of divergence was found between the southern and northern populations, such as Avaré and Uberlândia (*R*_*ST*_ = 0.912 / p = 0.00), Botucatu and Paranaiguara (*R*_*ST*_ = 0.670 / p = 0.01), and Botucatu and Uberlândia (*R*_*ST*_ = 0.636 / p = 0.00). While many of the comparisons with the population from Lençóis Paulista had high *R*_*ST*_ values (*e.g.*, *R*_*ST*_ = 1.00 between Lençóis Paulista and Paranaiguara), the results were not significant (p = 0.99) due to reduced sample size.

**Table 2:** Indices of inter-population variation (*R*_*ST*_) for pairwise comparisons of the *P. moratoi* study populations.

	PA^1^	IT	UB	SC	BR	BA	AV	LP
Ituiutaba	0.153	-						
Uberlândia	0.012	0.129	-					
São Carlos	0.314^2^ *	0.090	0.309*	-				
Brotas	0.366*	0.160*	0.365*	0.007	-			
Bauru	0.517*	0.340*	0.552*	0.163*	0.163*	-		
Avaré	0.933	0.701	0.912*	0.276*	0.152	0.358*	-	
Lençóis Paulista	1.000	0.607	0.922	0.033	-0.114	0.003	0.143	-
Botucatu	0.670*	0.188	0.636*	0.224	-0.220	-0.205	0.610*	0.507

1 PA = Paranaiguara-GO; IT = Ituiutaba-MG; UB = Uberlândia-MG; SC = São Carlos-SP; BR = Brotas-SP; BA = Bauru-SP; AV = Avaré-SP; LP = Lençóis Paulista-SP; BO = Botucatu-SP;

* = significant (p < 0.05).

Overall, 44 of the 122 distinct alleles produced by the neutral markers were exclusive to one of the populations. The southern populations were characterized by reduced numbers of private alleles, with four being recorded in Bauru, two in Avaré and Botucatu, and one in Lençóis Paulista. In the northern portion, by contrast, nine private alleles were recorded in Brotas, eight in Uberlândia, and seven in Paranaiguara and São Carlos.

**Figure 2:**
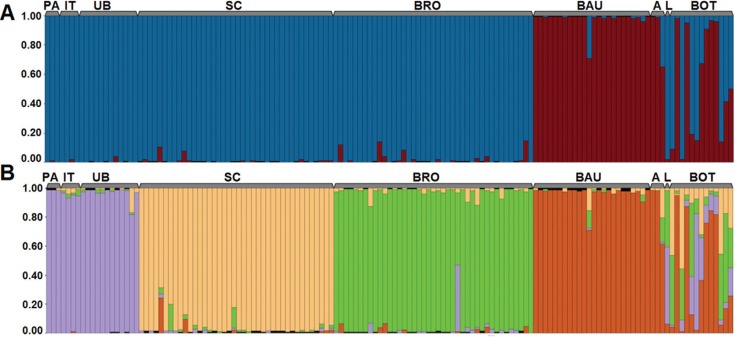
Population structure. Q plots were generated by the STRUCTURE program. These are based on a multilocus analysis of the nine *P. moratoi* populations. Each vertical line represents an individual, and the percentage of the different colored segments in each line is proportional to the adhesion of the individual to each of the *k* groups. (A) principal genetic structuring (*k* = 2), (B) weaker structuring (*k* = 4). PA = Paranaiguara; IT = Ituiutaba; UB = Uberlândia; SC = São Carlos; BRO = Brotas; BAU = Bauru; A = Avaré; L = Lençóis Paulista; BOT = Botucatu.

The STRUCTURE analysis identified two clusters representing the highest hierarchical level in the genetic structure of the *P. moratoi* populations (ΔK = 1083, Figure 2A). A more subtle subdivision assumed four clusters (ΔK = 534, Figure 2B). The CLUMMP analysis indicated that the convergence of the runs for both *k* values was extremely high (H' = 0.999 for *k* = 2 and H' = 0.999 for *k* = 4).

The principal structure of the data (*k* = 2) indicates a profound degree of structuring between the central (São Carlos and Brotas) + northern populations (Paranaiguara, Ituiutaba and Uberlândia) and the Bauru population, to the south, located on the left margin of the Tietê River (downstream flow). The other southern populations (Avaré, Lençóis Paulista, and Botucatu) presented a mixed distribution overlapping the two clusters (Q < 0.7). While the Botucatu population is characterized by an excess of empty genotypes, with few or no data being available for most individuals, it presented the same pattern of mixture with the geographically closest populations, that is, Avaré and Lençóis Paulista, which indicates an adequate amount of informative data. At *k* = 4, less profound structuring was detected, with the populations from Minas Gerais and Goiás forming a single cluster, three others clusters on the populations from São Carlos, Brotas, and Bauru, and the southern populations (Avaré, Lençóis Paulista, and Botucatu) once again presenting a mixed pattern, with a slight tendency to group with the Bauru population (see Figure 2B).

**Figure 3:**
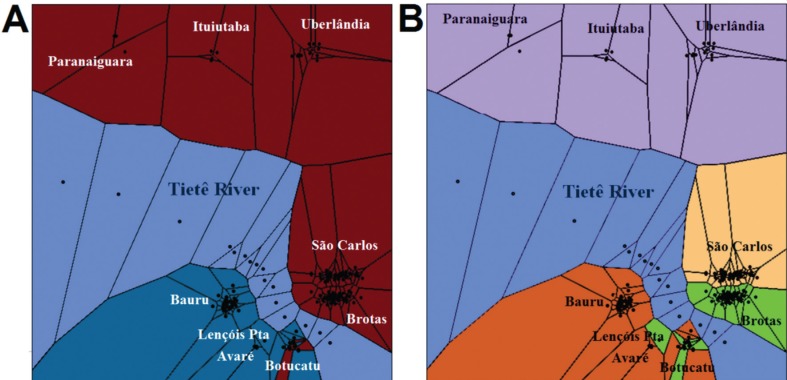
Spatial structure. Clusters produced by the TESS analysis, in which each individual is assigned to the group with which it shares most ancestrality (> Q). (A) *k* = 2, (B) *k* = 4. The dummy points, which are points where no population were sampled, represent the Tietê River (light blue). The same color scheme was used as for the STRUCTURE analysis (Figure 2) in order to facilitate comparisons.

Based on the initial STRUCTURE analyses, barriers to gene flow were added to the data input to represent the location of the Tietê River, which apparently represents the major barrier to the dispersal of *P. moratoi* populations. In this analysis, the plateau of the DIC values for the 10 independent runs was reached when *K*_*max*_ = 4, where *k* = 2 and *k* = 4 (Figure 3A, B) identified similar clusters to those detected by STRUCTURE. Following the correction by CLUMPP of the independent runs, the populations presented a high membership coefficient (Q > 0.7) for *k* = 2 (H' = 0.957), the exception being the individuals from the Botucatu population. At *k* = 4 (H' = 0.754), high Q values were observed in all the individuals from the Paranaiguara, Ituiutaba, and Uberlândia populations, all from the same cluster, Bauru and Avaré (also from the same cluster), and the population from Brotas. Mid-range Q values (0.5–0.7) were obtained for the São Carlos and Lençóis Paulista, and also for the Botucatu population, but in individuals representing distinct clusters. This analysis revealed an almost perfect differentiation between the population groups located on the left (southern) margin of the Tietê and the right (northern) margin of this river (Figure 3A, B). Exceptions were the Botucatu (at both *k* = 2 and *k* = 4) and Lençóis Paulista (*k* = 4) populations which had individuals that were assigned to different clusters.

The analysis of isolation by distance, based on the pairwise comparison of all the populations detected a significant tendency (*r* = 0.638; p < 0.002). However, when only the populations in the vicinity of the Tietê River (within a radius of 80 km) are included in the analysis, that is, excluding the populations from Minas Gerais and Goiás, the isolation by distance was not significant (*r* = −0.222; *p =* 0.804).

### Demographic analyses

The delta values of the BAYESASS runs were within the range expected for a pattern of convergence. Plots produced by TRACER indicated that the chain was well mixed (results not shown), confirming that the convergence of runs has been reached and that the estimates of gene flow were probably reliable. In general, contemporary migration rates (Supplementary Material Table S1) are low (*m*_c_ < 0.10), except for three significant one-way routes between Uberlândia and Ituiutaba (15% of migrants between populations 119 km apart), Uberlândia and Paranaiguara (10% of migrants, distance of 243 km), and Bauru and Avaré (12% of migrants, distance of 59 km). The highest rates of self-recruitment were found in the central populations, São Carlos and Brotas.

As for historic migration using the 95% confidence intervals (CI), the most significant routes (> 1%) were between Brotas and Bauru (0.017; CI:.0.000 – 0.037), Brotas and Uberlândia (0.016; CI: 0.000 – 0.035) and Brotas and São Carlos (0.016; CI: 0.000 – 0.036), and between São Carlos and Brotas (0.015; CI: 0.000 – 0.035). The largest population-scaled mutation rate was recorded for the northern population from Ituiutaba (Θ = 1.14; CI: 0.00 – 2.80), and the smallest, in the southern population from Avaré (Θ = 0.49; CI: 0.00 – 0.97). Based on the formula Θ = 4N_e_μ, the N_e_ for each population was 205 (CI: 0 – 525) for Paranaiguara, 224 (CI: 0 – 551) for Ituiutaba, 108 (CI: 0 – 433) for Uberlândia, 211 (CI: 0 – 524) for São Carlos, 216 (CI: 0 – 537) for Brotas, 118 (CI: 0 – 420) for Bauru, 96 (CI: 0 – 420) for Avaré, 211 (CI: 0 – 485) for Lençóis Paulista, and 146 (CI: 0 – 472) for Botucatu.

No significant divergence was detected between the *m*_*c*_ and *m*_*h*_ values for the populations and routes analyzed as a whole (r = −0.01, p = 0.64), although there was a marked tendency for positive values (*i.e.*, *m*_*c*_ > *m*_*h*_) except for the Brotas to São Carlos, São Carlos to Brotas, and Brotas to Bauru routes (*m*_*h*_ > *m*_*c*_). Similarly, no significant difference was found between contemporary and historic migration patterns in the northern (r = −0.28, p = 0.40, with a positive tendency) and southern populations (r = 0.25, p = 0.23, also with a positive tendency), although migration rates were significantly higher in the past in the central populations (r = 0.98, p = 0.03, with a negative tendency).

No evidence of recent decline was detected in any of the populations using either the one-tailed Wilcoxon test or the shifted mode, with the exception of the now extinct population from Botucatu. Significant results were obtained for both tests, using the TPM model with 70% and 90% SMM (Wilcoxon p = 0.05 and shifted mode = 11% of the rare alleles), and no significant bottleneck one-tailed Wilcoxon: p = 0.15) was detected in the case of the SMM model.

## Discussion

The populations located in the southern portion of the geographic distribution of *P. moratoi*, that is, on the left margin of the Tietê River, presented lower levels of genetic diversity in comparison with the other populations. While most of these southern populations are represented by small samples, which may explain part of this difference, it is important to note that the Bauru population, represented by more than 20 individuals, was characterized by low levels of diversity, which is typical of the region. In addition, despite the small size of the samples from the northern localities of Paranaiguara (n = 3) and Ituiutaba (n = 4), the fact that these populations presented high levels of diversity contradicted possible sampling effects.

It is interesting to note that some southern populations (*e.g.* Avaré and Lençóis Paulista, pers. obs.) show signs of drastic decline, while others (*i.e.*, Botucatu) have become extinct. This suggests that the reduced indices of diversity recorded in this region may be related to small populations sizes, a characteristic also observed in the estimates of historic population size. Inferences on immigration rates obtained in MIGRATE are generally not influenced by sample size, although the confidence interval tends to be larger in samples with fewer individuals (10 individuals per population – Beerli, 2004). However, the high N_e_ value recorded in a single southern population (Lençóis Paulista) may have been related to the small sample size (only one individual), which probably affected the reliability of the estimates provided by MIGRATE. Smaller populations tend to be more susceptible to extinction resulting from stochastic processes, which may explain the extinctions observed in the southern sector. Differences in inter-population diversity have also been recorded in the European tree-frog, *Hyla arborea*, which is not considered endangered in southeastern Europe, whereas the populations in the western portion of the distribution appear to be more vulnerable, with marked population decline and low levels of genetic diversity (Arens *et al.*, 2006; Dubey *et al.*, 2009).

In addition to its low genetic diversity, the Botucatu population also presented high levels of endogamy. Evidence of a recent bottleneck was also found in this population. Jim and Caramaschi (1980) analyzed specimens from this population and found only a limited amount of variation in traits such as body size and coloration. This was the only *in vivo* study of this population, however, and there is no unequivocal evidence of anomalies that might represent the effects of inbreeding depression, which may have contributed to the disappearance of this population by reducing the adaptive potential of the individuals. Nonetheless, the low level of genetic diversity found in the present study and the evidence for endogamy indicate that the population would probably not have been viable over the long term. In addition, as the population was located within an urban perimeter, it seems likely that it was effectively isolated from the others, leading to high rates of inbreeding. In fact, the area occupied by the species in Botucatu has been impacted by a series of anthropogenic processes, including the drainage of wetlands, introduction of exotic species and grazing animals, urbanization, and pollution (Rolim, 2009), all of which presumably contributed to the decline in *P. moratoi* numbers. This may be a premature conclusion, however, given the large proportion of missing genotypes resulting from the poor quality of the DNA in many specimens, as well as the existence of PCR inhibitors in these tissues. On the other hand, the genetic diversity recorded in this population was similar to that found in other southern populations (and was assigned to the same clusters), which does support the reliability of the few genotypes analyzed.

Despite the genetic divergence observed between the southern populations and those from the northern and central sectors of the species' distribution, the fact that the marker *Pmoratoi*μ5 was not amplified in the southern populations reinforces the differences encountered in the other analyses (*e.g.*, *R*_*ST*_, STRUCTURE, TESS). The Tietê River effectively separates the clusters (see TESS data), and its role in the structuring of the populations is reinforced by the non-significant isolation by distance found among the populations located in the vicinity of this river. An analysis of the morphological characteristics of the *P. moratoi* populations found little divergence, except for subtle differences in the coloration of the specimens (Costa, 2012). The specimens from Bauru are dark gray with black spots, and have a velvety sheen which distinguishes them from all other populations. Morphometrically, three groups were identified: Botucatu, Brotas + São Carlos, and Bauru + Avaré + Lençóis Paulista. This is similar to the *k* = 2 results obtained from the STRUCTURE and TESS programs, excluding the populations from Minas and Goiás, which were not included in the study. As for the differences in phenotypic traits, see Costa (2012), the employment of the hypervariable microsatellite markers detected considerable variation among populations, in contrast with Forti *et al.* (2016) who used a more conservative marker (the 16S gene).

The differentiation of the Botucatu population seen in this analysis was based on the much lower mean dimensions. Jim (2002) noted that the Botucatu region represents and “island” of colder climate related to its latitude and high altitude (> 800 m), where temperatures can reach below 0 °C (Engea, 1990). This feature of the region may have contributed to the unique morphometric characteristics of the local *P. moratoi* population (Costa, 2012).

The spatial location of the main barriers to gene flow, as identified by our analysis, closely matches the current course of one of the main rivers in the region. The genetic evidence indicates that the Tietê River constitutes a major barrier to the dispersal of these toads and may also have played an important zoogeographic role in the past, either as a river or associated geological features. In another species of the same genus, *P. boiei*, a phylogenetic analysis based on the molecular analysis of mitochondrial and nuclear sequences also detected a major genetic division between populations on opposite banks of the Tietê River (Amaro *et al.*, 2012). In a study of the herpetofauna of the Brazilian Amazon, Moraes *et al.* (2016) found that rivers are important barriers to dispersal, in particular for amphibians.

Two other major rivers also separate *P. moratoi* populations. One is the Grande River, which separates the Minas Gerais population from all others, and the Paranaíba River, which separates the Paranaiguara population in Goiás (see Figure 1). All these rivers are part of the Paraná basin (Souza *et al.*, 2005), and were formed during the upper Pleistocene (Stevaux and Santos, 1998). However, the two rivers in the northern sector of the species' range appear to have played a less prominent role in the structuring of the *P. moratoi* populations, suggesting these rivers might be either a relatively recent or a semi-permeable barrier.

The subtle subdivision (*k* = 4) found among nearby populations was expected given the philopatry of amphibians to their breeding sites and reduced vagility (Beebee, 2005). Together, these limit gene flow and may result in fine scale structuring, that is, even over distances of less than 10 km (Shaffer *et al.*, 2000; Lampert *et al.*, 2003). However, some species do have considerable dispersal capacity (Pyke and White, 2001; Funk *et al.*, 2005), even in areas with high levels of anthropogenic impact (Arruda *et al.*, 2011). In this case, susceptibility to fragmentation is highly species-specific and will depend on the vagility of the species, the permeability of the matrix, and historic population size and structure, all of which impede reliable generalizations (Wiens, 1997; Galbusera *et al.*, 2004; Cushman, 2006; Luoy *et al.*, 2007).

The reduced effective population sizes and diversity of the southern populations suggest that they are peripheral, based on the analyses and predictions of Lawton (1993), Vucetich and Waite (2003), and Garner *et al.* (2003). The demographic characteristics of peripheral populations are more likely to suffer impacts, especially from climatic extremes (Lesica and Allendorf, 1995).

The central populations (São Carlos and Brotas) presented the highest rates of self-recruitment and significantly lower contemporary gene flow in comparison with historic migrations (*m*_*h*_ > *m*_*c*_ / r = 0.98, p = 0.03, with a negative tendency), despite the reduced distance between them (22 km) and the absence of riverine barriers. The opposite pattern was found between other populations (*m*_*h*_ < *m*_*c*_). In the specific case of São Carlos, anthropogenic impact is the highest of any of the populations analyzed, and is characterized by a mosaic of sugarcane plantations (22% soil coverage) and areas of regenerated forest interspersed with small remnant islands of native vegetation (Rudorff *et al.*, 2004; Carvalho Jr *et al.*, 2010). Even in the southern areas, for example, soil coverage by sugarcane plantations is no more than 10% (Rudorff *et al.*, 2004). In fact, the Brazilian state of São Paulo, where the central sector is located, has the lowest level of protection of the Cerrado biome, with only 13% of the original vegetation remaining (Sano *et al.*, 2007). Agricultural fragmentation may increase random drift (Johansson *et al.*, 2007), affecting both neutral genetic variability and adaptive traits. However, few amphibian studies have focused on the role of anthropogenic processes, such as urbanization and agriculture, which generally (see exceptions Youngquist and Boone, 2014; Rodríguez *et al.*, 2015) reduce vagility, genetic diversity (*e.g.*, Johansson *et al.*, 2005; Noël *et al.*, 2007; Arens *et al.*, 2007), survival rates and geographic distribution (Raxworthy and Nussbaum, 2000; Vallan, 2002), with an overall increase in landscape resistance (Goldberg and Waits, 2010).

Our study indicates that *P. moratoi* is vulnerable to anthropogenic processes, given that the removal of the native vegetation for the implantation of plantations in the central sector has contributed to the reduction in the dispersal of these toads (*m*_*h*_ > *m*_*c*_). On the other hand, it is also possible to infer that, as the central and northern populations are larger, they are less vulnerable to extinction resulting from short-term stochastic processes, in contrast with the Botucatu population, which is now extinct. Levels of genetic diversity appear to persist due to the relatively recent process of population fragmentation, although with ongoing isolation and resulting increase in genetic drift over the long term, a loss of genetic diversity and increase in endogamy will be expected, especially in the central populations.

### Implications for conservation

Based on the extension of the geographic distribution of *P. moratoi* (> 70,000 km^2^), and our findings on effective population size (approximately 1,500 individuals) and the number of adult individuals (< 250) in each population, we recommend the reclassification of the conservation status of the species according to the relevant IUCN (2016) criteria. Rather than Critically Endangered (CR), based on criterion B1ab (iii, v), we recommend the reclassification of *P. moratoi* as Vulnerable (VU), in accordance with criteria A2a and C2a (i). In this case, while less preoccupying, the species is still considered to be under some threat of extinction, given the evidence of decline in the southern populations and the reduced numbers of individuals.

The anuran fauna of the Cerrado is still very poorly known (Diniz-Filho *et al.*, 2004), and it seems likely that additional surveys will reveal the presence of additional populations, especially in the northern portion of the range of *P. moratoi*, where relatively high levels of genetic diversity were found.

Notwithstanding, the unique genetic characteristics of the southern populations and their reduced levels of genetic diversity associated with small numbers, endogamy, and population decline, all reinforce the need for high priority conservation measures in this region. In addition to increasing the probability of extinction through stochastic processes, reduced genetic diversity in amphibians has been associated with reduced fitness and neutral diversity (Andersen *et al.*, 2004; Lesbarrères *et al.*, 2005), which further reinforces our preoccupation with the southern *P. moratoi* populations.

The sum of the evidence presented here indicates the need for three principal conservation measures: (i) the definition of two management units, one consisting of the southern populations, on the left margin of the Tietê River in São Paulo, and the other of all the other populations. This primary division was reinforced by STRUCTURE (*k* = 2), which re-emphasizes the need for the separate management of these two units (Moritz and Faith, 1998); (ii) due to the levels of population decline and extinction found in the southern populations, we recommend that conservation management policies focus on the protection of habitats, especially through the creation of reserves (state and federal) and the promotion of connectivity, supported by demographic and genetic monitoring; and (iii) the collection of data on the fitness of these populations, based on molecular markers and phenotypic traits.

As the present analyses do not provide estimates of the divergence times of the different management units, we would recommend the application of a more systematic phylogeographic approach based on additional molecular markers (nuclear and mitochondrial) for a more reliable definition of the patterns and the timing of the diversification of the different lineages. We would also recommend the establishment of *ex situ* populations with individuals from the southern localities (as per the suggestion of Woodworth *et al.*, 2002), based on appropriate management measures (*i.e.*, maintenance of levels of genetic diversity, prevention of endogamy, and minimization of adaptations to captivity) with the aim of preserving these unique genetic stocks. The southern populations may be at the limit of viability due to the marked effects of drift, the reduced capacity to respond to selective pressures, and the low probability of any increase in genetic variability due to a rescue effect from other populations.

## Supplementary material

The following online material is available for this article:

Table S1 - Estimates of recent and historic migration.
