# Supplementary material for: Genetic diversity of Morato's Digger Toad, Proceratophrys moratoi: spatial structure, gene flow, effective size and the need for differential management strategies of populations
Source: Genet Mol Biol. 2017 Jun 5;40(2):502–14. doi: 10.1590/1678-4685-gmb-2016-0025 (PMC5488452; doi:10.1590/1678-4685-gmb-2016-0025)
Supplement: Supplementary file 1 [file 1415-4757-gmb-1678-4685-GMB-2016-0025-Suppl01.pdf]

Table S1 - Estimates of recent (m_c – mean of five runs, upper table) and historic (m_h, lower table) migration between pairs of populations using the BAYESASS and MIGRATE programs, respectively. The confidence interval for each value is shown in parentheses. High migration rates (≥ 10% for recent migrants and ≥ 1% for historic migrants) are underlined. The values in bold type represent the proportion of resident individuals per generation (i.e., self-recruitment, upper table) and theta values ($\Theta = 4N_e\mu$, lower table) in each population.

	Recent migration (BAYESASS) from								
to	PA[1]	IT	UB	SC	BR	BA	AV	LP	BO
PA	**0.715 (0.04)**	0.026 (0.02)	0.100 (0.06)	0.031 (0.03)	0.026 (0.02)	0.025 (0.02)	0.026 (0.02)	0.026 (0.02)	0.026 (0.02)
IT	0.022 (0.02)	**0.697 (0.03)**	0.146 (0.05)	0.022 (0.02)	0.022 (0.02)	0.022 (0.02)	0.022 (0.02)	0.022 (0.02)	0.022 (0.02)
UB	0.016 (0.02)	0.016 (0.02)	**0.866 (0.04)**	0.021 (0.02)	0.017 (0.02)	0.016 (0.02)	0.016 (0.02)	0.016 (0.02)	0.016 (0.02)
SC	0.007 (0.01)	0.007 (0.01)	0.007 (0.01)	**0.944 (0.02)**	0.008 (0.01)	0.007 (0.01)	0.007 (0.01)	0.007 (0.01)	0.007 (0.01)
BR	0.007 (0.01)	0.007 (0.01)	0.008 (0.01)	0.010 (0.01)	**0.941 (0.02)**	0.007 (0.01)	0.007 (0.01)	0.007 (0.01)	0.007 (0.01)
BA	0.010 (0.01)	0.010 (0.01)	0.010 (0.01)	0.010 (0.01)	0.011 (0.01)	**0.919 (0.02)**	0.010 (0.01)	0.010 (0.01)	0.010 (0.01)
AV	0.025 (0.02)	0.025 (0.02)	0.025 (0.02)	0.025 (0.02)	0.031 (0.03)	0.119 (0.05)	**0.701 (0.03)**	0.025 (0.02)	0.025 (0.02)
LP	0.031 (0.03)	0.031 (0.03)	0.032 (0.03)	0.031 (0.03)	0.067 (0.04)	0.032 (0.03)	0.031 (0.03)	**0.713 (0.04)**	0.031 (0.03)
BO	0.022 (0.02)	0.017 (0.03)	0.044 (0.04)	0.056 (0.05)	0.061 (0.05)	0.072 (0.05)	0.018 (0.02)	0.017 (0.02)	**0.693 (0.02)**
	Historic migration (MIGRATE) from								
to	PA	IT	UB	SC	BR	BA	AV	LP	BO
PA	**1.041 (1.62)**	0.005 (0.02)	0.005 (0.03)	0.006 (0.03)	0.007 (0.03)	0.005 (0.02)	0.005 (0.02)	0.008 (0.03)	0.005 (0.02)
IT	0.005 (0.02)	**1.140 (1.66)**	0.006 (0.03)	0.007 (0.03)	0.006 (0.03)	0.005 (0.02)	0.004 (0.02)	0.004 (0.02)	0.005 (0.02)
UB	0.004 (0.02)	0.004 (0.02)	**0.550 (1.65)**	0.008 (0.03)	0.016 (0.04)	0.005 (0.02)	0.005 (0.02)	0.004 (0.02)	0.004 (0.02)
SC	0.004 (0.02)	0.003 (0.02)	0.004 (0.02)	**1.070 (1.59)**	0.016 (0.04)	0.005 (0.02)	0.004 (0.02)	0.004 (0.02)	0.004 (0.02)
BR	0.004 (0.02)	0.004 (0.02)	0.005 (0.02)	0.015 (0.03)	**1.100 (1.63)**	0.007 (0.03)	0.004 (0.02)	0.004 (0.02)	0.005 (0.02)
BA	0.004 (0.02)	0.004 (0.02)	0.004 (0.02)	0.007 (0.03)	0.017 (0.04)	**0.600 (0.60)**	0.004 (0.02)	0.004 (0.02)	0.004 (0.02)
AV	0.004 (0.02)	0.004 (0.02)	0.005 (0.02)	0.005 (0.03)	0.008 (0.03)	0.006 (0.03)	**0.490 (1.64)**	0.005 (0.02)	0.005 (0.03)
LP	0.006 (0.03)	0.005 (0.02)	0.007 (0.03)	0.010 (0.03)	0.006 (0.03)	0.007 (0.03)	0.006 (0.03)	**1.070 (1.38)**	0.009 (0.03)
BO	0.006 (0.03)	0.005 (0.03)	0.006 (0.03)	0.006 (0.03)	0.008 (0.03)	0.005 (0.02)	0.005 (0.02)	0.005 (0.02)	**0.740 (1.67)**

[1]PA = Paranaiguara; IT = Ituiutaba; UB = Uberlândia; SC = São Carlos; Brotas; BA = Bauru; AV = Avaré; LP = Lençóis Paulista; BO = Botucatu.
